# Pannexin-1 Channels as Mediators of Neuroinflammation

**DOI:** 10.3390/ijms22105189

**Published:** 2021-05-14

**Authors:** Joon Ho Seo, Miloni S. Dalal, Jorge E. Contreras

**Affiliations:** 1 Department of Neurology and Nash Family, Department of Neuroscience, Friedman Brain Institute, Icahn School of Medicine, Mount Sinai, New York, NY 10029, USA; joonho.seo@mssm.edu; 2 Department of Pharmacology, Physiology and Neuroscience, New Jersey Medical School, Rutgers University, Newark, NJ 07103, USA; msd199@gsbs.rutgers.edu; 3 Department of Physiology and Membrane Biology, University of California Davis, Davis, CA 95616, USA

**Keywords:** neuroinflammation, Pannexin-1, hemichannel, brain injury

## Abstract

Neuroinflammation is a major component of central nervous system (CNS) injuries and neurological diseases, including Alzheimer’s disease, multiple sclerosis, neuropathic pain, and brain trauma. The activation of innate immune cells at the damage site causes the release of pro-inflammatory cytokines and chemokines, which alter the functionality of nearby tissues and might mediate the recruitment of leukocytes to the injury site. If this process persists or is exacerbated, it prevents the adequate resolution of the inflammation, and ultimately enhances secondary damage. Adenosine 5′ triphosphate (ATP) is among the molecules released that trigger an inflammatory response, and it serves as a chemotactic and endogenous danger signal. Extracellular ATP activates multiple purinergic receptors (P2X and P2Y) that have been shown to promote neuroinflammation in a variety of CNS diseases. Recent studies have shown that Pannexin-1 (Panx1) channels are the principal conduits of ATP release from dying cells and innate immune cells in the brain. Herein, we review the emerging evidence that directly implicates Panx-1 channels in the neuroinflammatory response in the CNS.

## 1. Background

**Figure 1:**
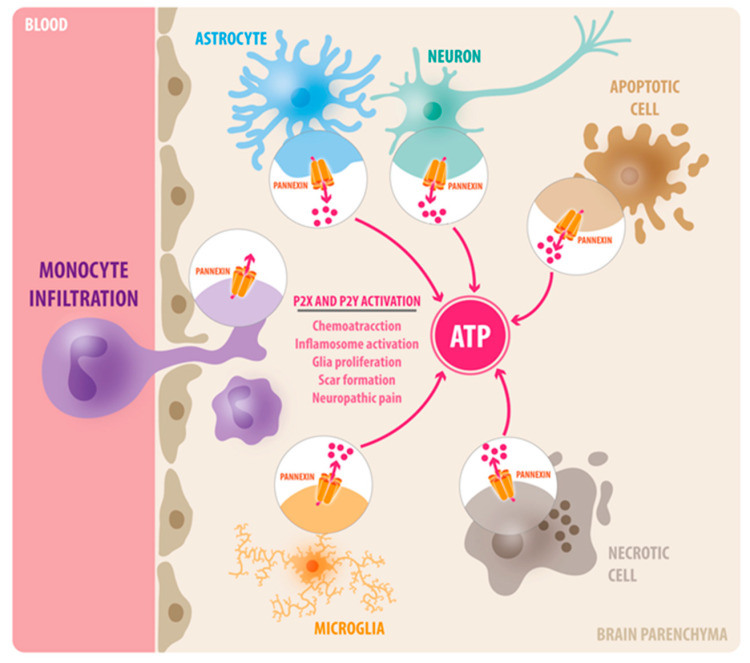
Diverse function of Panx1 channels in neuroinflammation. In the brain, Panx1 channels are expressed in neurons, astrocytes, and microglia. Upon tissue injury, Panx1 channels are activated and become permeable to ATP, which acts as a central damage-associated molecular pattern (DAMP) via P2X and P2Y receptors. In consequence, extracellular ATP initiates a complex cascade of inflammatory response, including immune cell infiltration, inflammasome activation, glia proliferation, and scar formation.

Over the past few decades, rapid advancements in medical research have led to increasing life expectancy, but the socioeconomic impact of diseases associated with aging, including neurodegenerative diseases, traumatic brain injury (TBI), and stroke, is increasing considerably [1,2,3]. While the pathological mechanisms of the aforementioned diseases are not completely understood, it is becoming clear that neuroinflammation is a shared pathophysiological feature [4,5,6,7,8]. In general, inflammation is an adaptive immune response initiated by noxious stimuli such as infection or tissue injury [9]. While a successful inflammatory response results in wound healing and elimination of invading pathogens, persistent and uncontrolled inflammation may contribute to negative outcomes, including the exacerbation of cell death and tissue damage [10]. The native immune response triggered by brain pathologies can also be beneficial or detrimental depending on the timing of the resolution for the inflammatory response. The identification and understanding of the molecular mediators responsible for neuroinflammation may contribute to the development of novel therapeutic targets for numerous neurological diseases. Here, we focus on the role of Pannexin-1 (Panx1) channels as promoters of inflammation in brain pathologies. Panx1 proteins are expressed in most brain cells, including neurons and glia, from early developmental stages to throughout adulthood [11,12,13,14,15,16,17,18,19,20,21]. They work as pore conduits for adenosine triphosphate (ATP) release, which consequently activates purinergic signaling via P2X and P2Y receptors [22]. Activation of Panx1 channels has been implicated in chemotaxis [23], leukocyte infiltration [24], inflammasome activation [25] and pyroptosis [26], and neurophatic pain [27,28] (Figure 1).

## 2. The Innate Immune System of the Brain and ATP Signaling

As inflammation involves the orchestration of different cell-types, addressing Panx1 signaling in neuroinflammation posits an understanding of the contribution of many cells, including the neurons and glia; the two main types of cells in the brain [29,30]. While neurons transmit electrical signals from one cell to another; glial cells, including microglia, astrocytes, oligodendrocytes, and ependyma cells, maintain homeostasis of the brain parenchyma and regulate neuronal activity [31]. Microglia and astrocytes are recognized as essential parts of the innate immune system of the brain. They initiate complex interrelated inflammatory responses upon injury or infection [10]. Thus, most recent research in neuroinflammation has been focused on understanding the activation status of these cells and the application of this knowledge for exploring therapeutic approaches for neurodegenerative diseases, stroke, and TBI [8,29].

Microglia are brain-resident macrophages that continuously survey their local environment in the brain parenchyma. These cells are usually the first cells to respond to injury or insult to the brain [32]. During development, microglia originate from primitive macrophages from the embryonic yolk sac that invade the brain parenchyma and proliferate [33]. In a steady-state, microglia are prompt to sense inflammatory molecules [34], promote neuronal survival [35], participate in activity-dependent synaptic remodeling [36], and phagocytose particles from damaged cells [37]. Once they sense inflammatory signals, microglia become activated, which leads to retraction of their processes, hypertrophy of the cell body, and proliferation [37]. During the early phase of tissue damage or infection, microglia rapidly extend their processes towards the injury or infected site [37]. These intricate processes are mediated by purinergic signaling, via nucleotides release (i.e., ATP or UDP), and the activation of P2Y6, P2Y12, and P2X4 receptors [37,38]. It is thought that this early and rapid response enables microglia to assess and prevent further damage [38,39]. Once at the site of injury, microglia begins to resolve the inflammation, mainly by the phagocytosis of cellular debris and dead cells [40]. These processes are also facilitated by purinergic signaling, mainly by P2Y6-mediated detection of UDP released from dying cells [41]. It has been demonstrated that focal brain injury recruits microglia that phagocytose cellular debris and form a honeycomb network around glial-limitans, and that antagonism of these processes worsens brain damage [39]. Similarly to reactive astrocytes, activated microglia secrete numerous pro- and anti-inflammatory cytokines (i.e., TNF-α, IL-1β, IL-6, and TGF-β), as well as chemokines (CCL2, CCL3, CCL4, CXCL1, and CXCL4), which promotes infiltration of peripheral leukocytes into the brain. In addition, microglia also produce and release metalloproteinases, nitric oxide, and reactive oxygen species, all of which can cause damage to neurons and the blood–brain barrier and, consequently, further enhance recruitment of immune cells [42,43]. While multiple studies have indicated that activated microglia are neurotoxic, it is important to note that microglia can also promote neuronal survival by releasing neurotrophic factors such as brain-derived neurotrophic factor (BDNF) and neutrophin-3 (NT-3) [44,45,46,47]. The neuroprotective role of microglia, however, is not addressed in detailed in this review because there is not yet enough evidence that connects it with Panx1 channels.

Astrocytes are parenchymal glial cells that are ubiquitous in the central nervous system (CNS), where they constitute the most abundant population of cells. They are involved in numerous and essential physiological and pathological processes [48]. In the healthy CNS, astroglia provide structural support to neurons and separation from the parenchymal structures, including the meninges and blood vessels [49]. They play key roles in glucose metabolism, blood flow regulation, and synapse development and plasticity [50]. Astrocytes also maintain brain homeostasis by balancing ions, extracellular fluids, and neurotransmitters. On the other hand, similar to microglia, astrocytes can respond to various damage-associated molecular patterns (DAMPs) and pathogen-associated molecular patterns (PAMPs), including extracellular ATP and a variety of pro-inflammatory cytokines, such as IL-1β, IL-6, and IFN-γ [51]. Astrocytes then become “reactive”, which can lead to cellular hypertrophy, proliferation, and migration [52]. While the beneficial effects of reactive astrogliosis during CNS injury include wound healing, neuronal protection, and repair of the blood–brain barrier [10], sustained reactive astrogliosis can lead to harmful effects. The excessive production of pro-inflammatory chemokines and cytokines ultimately interferes with axonal growth and synapse formation [52]. The release of chemokines and cytokines is directly involved with the recruitment and activation of monocytes, macrophages, T lymphocytes, dendritic cells, and neutrophils. Studies using various astrocyte specific transgenic mouse lines revealed that the manipulation of reactive astrocytes can lead to profound changes in disease outcomes in different inflammatory CNS diseases, including TBI, microbial infection, autoimmune inflammation, and neurodegenerative diseases [52]. For example, astrocyte specific deletion of the CCL2 chemokine showed a less severe autoimmune encephalomyelitis that correlated with reduced infiltration of macrophages and T lymphocytes and, subsequently, an attenuated inflammatory response [53]. Consistently, prevention of a reactive phenotype in astrocytes by simultaneous deletions of IL-1α, TNF-α, and C1q significantly extended survival in an ALS mouse model [54].

Extracellular ATP release and purinergic receptors are important molecular players in the pathophysiological functions of reactive astrocytes. Indeed, reactive astrocytes are associated with several brain pathologies, including Alzheimer’s disease, brain ischemia, multiple sclerosis, and neuropathic pain [55,56,57]. Among those channels that permit extracellular ATP release in astrocytes, connexin-43 (Cx43) hemichannels are probably the most studied. It has been proposed that Cx43 hemichannels are required for astrocyte migration and glial scar formation [51,58]. While astrocytic Panx1 channels are less understood in the context of neuroinflammation, they have been shown to be important in the generation and spread of the calcium wave, which is used by astrocytes as mean of intercellular communication [59,60].

## 3. Neuroinflammation and Extracellular ATP

As mentioned above, cellular damage in the brain often initiates complex cascades of inflammatory response that start with: (1) the release of PAMPs and DAMPs from damaged neuron and glia, (2) activation of brain resident microglia and astrocytes, (3) production of chemokines and cytokines, and (4) recruitment of peripheral cells including leukocytes to the site of injury [29]. One of the key mediators that is released from damaged and dying cells to the extracellular milieu during inflammation is ATP [61]. Extracellular ATP has been shown to serve as a danger signal that mediates inflammation by several pathways, including activation of inflammasomes [62] and induction of immune cell infiltration [63]. ATP acts as a central “find-me” signal to attract monocytes, macrophages, and microglia (in the CNS) to the site of injury [26]. Moreover, extracellular ATP has been shown to be directly involved in inflammasome activation via P2X7 receptors, which subsequently leads to the efflux of potassium and activation of the nucleotide-binding oligomerization domain receptors (NLR), pyrin domain-containing 3 (NLRP3) [25]. Uncontrolled activation of inflammasome can result in inflammation-driven cell death, termed pyroptosis, which can spread through the tissues and could be one of the mechanisms underlying secondary damage [64]. In brain trauma, inflammasomes contribute to unfavorable outcomes; for example, in rodent models of TBI, it has been shown that the major inflammasome proteins NLRP3, ASC, and Caspase-1 are upregulated in neurons, microglia, and astrocytes [65]. Targeting therapies against inflammasome proteins including NLRP1, NLRP3, ASC, or Caspase-1 reduced the innate inflammatory response and tissue damage in rats after TBI [66]. Importantly, IL-1β, a major product of the activated inflammasome, has also been shown to play a pivotal role in triggering and sustaining TBI-induced inflammatory processes. It has been directly linked to other rodent models of brain injury, including ischemia, encephalitis, and age-related cognitive impairment [67,68,69].

In addition, elevation of extracellular ATP has been associated with neuronal death in hypoxic/ischemia [70], tumor cell death [71], and pyroptosis [72]. The best described mechanism of ATP release is via the exocytosis of granules filled with ATP, as shown in pancreatic cells, neurons, platelets, and mast cells [73,74,75,76,77]. However, over the past decade, Panx1 channels have been identified as an effective mechanism for ATP release in various inflammatory conditions that affect the CNS, including in models of TBI [24], cerebral ischemia [78,79], and epilepsy [80,81].

## 4. Pannexin-1 (Panx1) Channels

**Figure 2:**
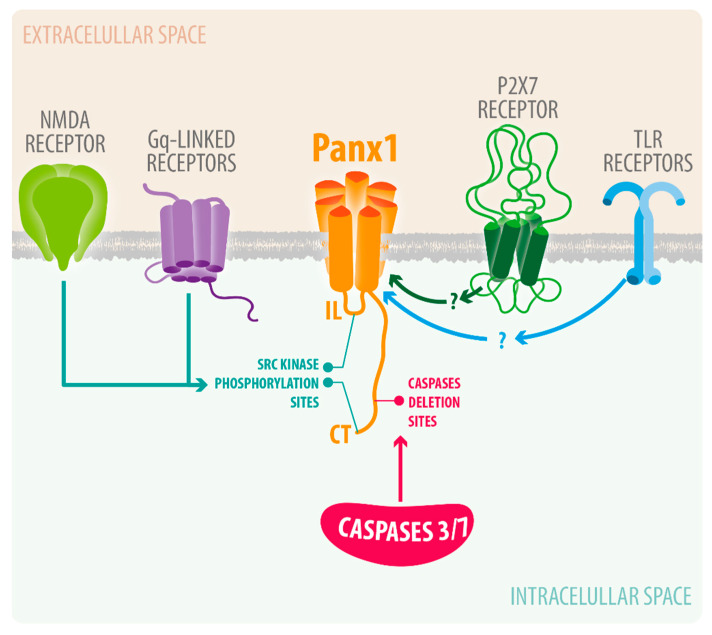
Current understanding of Panx1 channel activation in neuroinflammation. Upon cerebral ischemia, NMDA receptors activate Panx1 channels via phosphorylation by Src Kinase. Various infections and injuries can activate P2X7 and TLR receptors, which have been shown to activate Panx1 channels.

Pannexin channels are a group of transmembrane channel proteins responsible for autocrine/paracrine communication. They were discovered as proteins homologous to innexins found in invertebrates, which form gap junction channels. There are three gene family members of pannexin channels expressed in mammals, namely Panx1, Panx2 and Panx3 [11]. Panx1 is ubiquitously expressed in various organs and tissues, including the brain, spinal cord, eye, heart, and thyroid [11]. Panx2 and Panx3 expression are more restricted to certain tissues; for instance, Panx2 is highly expressed in the brain and Panx3 is mainly expressed in skin and bone Based on the structural properties of these channels, they allow movements of ions, small metabolites, and secondary messengers up to 1 kDa in size [82]. The pannexin channels have been found to play important roles in normal development and various physiological functions, including skin and bone development, synaptic plasticity, and blood vessel regulation [22,78,83,84,85,86]. Of all pannexins, Panx1 proteins are the most abundantly expressed in the brain, and comprising various cell-types, such as microglia, astrocytes, oligodendrocytes, and neurons [11,12,13,14,15,16,17,18,19,20,21]. An important function of Panx1 channels is associated with calcium signaling via ATP release and the activation of P2X and P2Y receptors [87]. It has been shown that the binding of ATP to G-coupled P2Y receptors will activate PLC and IP3 pathways, which release calcium from intracellular store (ER) [87]. It has also been proposed that an increase of calcium concentration from intracellular sources further activates Panx1 channels mediating an ATP-dependent ATP release, which further propagates calcium signaling to neighboring cells [22]. Moreover, Panx1 channels have been shown to form a complex with P2X7 purinergic receptors [88]; the latter are known to be molecular players that amplify CNS damage and neuroinflammation in various brain pathologies [89]. Various other plasma receptors or proteins involved brain pathologies, including NMDA receptors or caspases, have been linked to Panx1 channel activation (Figure 2). Below we described some of them in further detailed.

## 5. Pannexin-1 Channels in Neuroinflammation

Panx1 channel activation is broadly associated with pathological processes including cancer [90,91], ischemia [78,92], platelet activation [93], seizure [94], immune cell migration [83], and HIV viral replication [95]. Freeman and colleagues showed that reducing Panx1 protein levels with shRNA, or blockade of Panx1 channel activity using carbenoxolone or probenecid, significantly reduced the level of cell growth and cell migration of A375-P and A375-MA2 melanoma cell lines [90]. Furthermore, Panx1 channel activation is implicated in seizure activity in humans and animal models of epilepsy [94,96]. Using post-operative human brain samples, Dossi and her colleagues demonstrated that pharmacological inhibition of Panx1 channel with probenecid and mefloquine blocked ictal discharges. In addition, the authors showed that mice harboring genetic deletion of Panx1 were resistant to kainic acid-induced seizures [94,97]. Panx1 channels have also been shown to play an important role in CD4+ T lymphocytes during the course of infection by HIV-1. By using fluorescent techniques, it was shown that HIV isolates (R5 and X4) induced transient-early and sustained-later opening of Panx1 channels, which was dependent on HIV binding to the CD4/CCR5/CXCR4 complex [95].

Since many, if not all, CNS injuries and diseases involve a certain degree of neuroinflammation [98], it is likely that the therapeutic modulation of the neuroinflammatory response may serve as a potential approach for treating brain injuries and neurological diseases. Indeed, many novel therapeutic targets for neurodegenerative diseases that were traditionally thought to be neuronal in nature, including Alzheimer’s disease (AD), Parkinson’s disease (PD), and amyotrophic lateral sclerosis (ALS), modulate the immune response of astrocytes and microglia [52,99,100,101,102,103]. Over the past decade, the Panx1 channel has been explored as a target in various brain pathologies (see Table 1). Below, we focus on specific CNS pathologies where Panx1 channels have been reported to play a role in tissue injury and the neuroinflammatory response.

### 5.1. Spinal Cord Injury, Neuropathic Pain, and Orofacial Pain

The neuroinflammatory response produced by spinal cord or nerve injury has been recently associated with Panx1 channel activation in astrocytes and microglia [104,105,106]. Garre et al. showed that the inflammatory action of the fibroblast growth factor-1 (FGF-1) is mediated by the opening of both Cx43 hemichannels and Panx1 channels in astrocytes from spinal cord slices. This response is first initiated by activation of the astrocytic Panx1 channel, which leads to an increase in membrane permeability to small molecules, elevated levels of intracellular calcium, and ATP release [107]. FGF-1-induced Panx1 activation then promotes the activation of microglia, enhancing the release of pro-inflammatory cytokines through a mechanism depending on P2X7 receptors. The authors suggested this circuit favors the recruitment of leukocytes into the injured spinal cord impacting negatively on neuronal function and survival. Consistently, a sole study has also shown that blockade or global deletion of Panx1 channels reduced spinal cord inflammatory lesion in a mice model of experimental autoimmune encephalomyelitis (EAE). The global deletion of Panx1 also delayed clinical signs of EAE and decreased mortality when compared to wild type animals. The specific role of different cell types expressing Panx1 in this model, however, remains to be established [108].

The negative effects of opioid withdrawal and neuropathic pain that affect the spinal cords normal function have been recently associated with the activation of microglia Panx1 channels [109]. The group of Trang has shown that blockade of Panx1 channels or specific deletion of microglial Panx1 channels alleviates morphine withdrawal, likely via the inhibition of ATP release. It is established that withdrawal from morphine induces long-term synaptic facilitation in specific neuronal layers of the spinal dorsal horn that mediate opiate analgesia. Pharmacological blockade of the Panx1 channel or microglia-specific Panx-1 deletion prevent the synaptic facilitation mediated by morphine withdrawal. The same group later demonstrated that deletion of microglial Panx1 channels also alleviated the joint pain caused by mechanical allodynia. [109]. The authors proposed that the purinergic receptor P2X7, an established mediator of joint pain caused by intra-articular injection of monosodium iodoacetate, activates panx1 in spinal microglia cells. Panx1 channel activation leads to release of the pro-inflammatory cytokine IL-1β, which is an important mediator of the neuroinflammatory response [109]. Consistently, the Bayliss group showed that deletion of Panx1 from myeloid cells (microglia and infiltrating monocytes) or treatment with Panx1 channel blockers attenuated early development of hypersensitivity to tactile and thermal stimuli induced by two types of nerve injury [106]. Interestingly, the author proposed that myeloid Panx1 channel activation in nerved injury-induced pain relies on Panx-1 protein phosphorylation at residue Y198A. This was supported by the experiments where transplantation of bone marrow cell expressing mutant Panx1-Y198A failed to rescue mechanical allodynia when compared to those transplanted with wild-type Panx1 [106]. Since Panx1 channels are phosphorylated at residue Y198 by a number of G_q_-linked receptors, the authors suggested that G_q_-linked receptors implicated in neuropathic pain, such as P2Y, histamine, and metabotropic glutamate receptors, could play a role in the activation of Panx1 channels in myeloid cells [106].

It has previously been shown that Panx1 channels are expressed in high levels in satellite glial cells (SGC) and trigeminal ganglion cells (TGC) [110]. Since the trigeminal nerve sends pain signals from the craniofacial areas to the central nervous system [111], it is plausible that Panx1 may be involved in these processes. Indeed, there is evidence that Panx1 channels may play a role in orofacial pain and migraine with visual or sensory aura [112,113]. Hanstein and colleagues demonstrated that, using a mouse model of chronic orofacial pain and astrocyte or neuron-specific Panx1 knock out mice, Panx1 channels in astrocytes and neurons contribute to tactile hypersensitivity [113]. More specifically, they showed that while deletion of Panx1 in GFAP-positive astrocytes completely prevented hypersensitivity, deletion of Panx1 in neurons reduced baseline sensitivity and the period of hypersensitivity [113].

Cortical spreading depression (CSD) is a phenomenon where slowly propagating waves of depolarization are followed by low brain activity [114]. CSD is thought to be the main cause of migraines with visual and auditory aura by activating perivascular trigeminal nerves [115,116,117,118]. Karatas and colleagues demonstrated that CSD caused neuronal Panx1 channel opening and caspase-1 activation, which was followed by the release of inflammatory molecules such as high-mobility group box 1 (HMGB1). They showed that cleaved caspase-1 and HMGB1 are only found in neurons where Panx1 channels are activated. The authors suggested that the inflammatory response triggered by opening of neuronal Panx1 channels further lead to astrocyte activation via the NF-κB pathway [92]. CSD is known to cause elevation in blood flow in the middle meningeal artery (MMA), followed by the dialation of MMA [119]. Treatment with the Panx1 inhibitor, carbenoxolone, completely inhibited MMA dilation, suggesting that Panx1 channels could contributed to CSD-induced trigeminal nerve activation [112]. Together, the evidence suggests that Panx1 channels are important conduits in the transmission of pain, and that they may represent a novel therapeutic target for different kinds of pain, including neuropathic pain, migraine with aura, and orofacial pain.

### 5.2. Brain Ischemia

Stroke is a cerebrovascular event where blood flow is blocked in a particular region of the brain, leading to cell death and inflammation [8]. Stroke is one of the five leading causes of death in the United States, and it causes major disability in survivors [120]. It can be categorized into two large categories, ischemic and hemorrhagic; ischemic stroke being by far the most prevalent, accounting for around 87% of all strokes [121]. In ischemic stroke, blood supply is obstructed by fatty buildups, called plaques. Ischemic stroke initiates a complex series of molecular events that lead to necrosis and apoptosis, followed by subsequent neuroinflammation [122].

With the acute cessation of blood supply and oxygen after an ischemic stroke, neurons in the affected area experience a catastrophic energetic failure, because aerobic production ATP is halted while the consumption remains constant [120]. Reduction in intracellular ATP induces depolarization of membrane potential leading to the activation of various ion channels, which results in disruption of ionic homeostasis [123]. Neuronal Panx1 channels, first demonstrated by Thompson and his colleagues, have been shown to activate early on after oxygen glucose deprivation in an in vitro model of ischemia [92]. As shown in Figure 2, they further showed that the neuronal Panx1 channel, the N-Methyl-D-Aspartate receptor (NMDAR), and the sarcoma (Src) kinase form a signaling complex that mediates neuronal death during anoxia-induced excitotoxicity in vitro and in vivo [78,92]. They concluded that the opening of Panx1 channels may be the major contributor to the dysregulation of ionic fluxes that leads to neuronal necrosis after ischemia [78,79,92].

In response to ischemic insult, neuronal necrosis and debris can activate microglia and astrocytes, which initiate the secondary neuroinflammatory events, including leukocyte infiltration. One of the key inflammatory molecules produced and released by microglia and astrocytes is IL-1β, which has a wide range of inflammatory effects [124]. IL-1β is mainly produced by inflammasome complexes that involve NLRP3, ASC, NEK7, and caspase-1 proteins [25]. A key signaling pathway that could lead to inflammasome activation is extracellular ATP acting on P2X7 receptors [125]. The evidence suggests that activation of Panx1 channels by injury increases the extracellular levels of ATP, which feeds back on P2X 7 receptors. This feed forward reaction triggers potassium efflux via P2X 7, which induces the formation of an active inflammasome complex [25]. While it is possible that the Panx1-P2X 7 signaling complex drives inflammation and secondary damage after ischemic injury, current data are conflicting. While some lines of evidence indicate that inhibition of Panx1 channels leads to positive outcomes after ischemia, others suggest that Panx1 channels do not contribute to injury. For example, Bargiotas and colleagues found that genetic deletion of Panx1 protein did not protect from middle cerebral artery occlusion (MCAO) in a mouse model of cerebral ischemia [126]. However, a double knockout mouse line, were both Panx1 and Panx2 were deleted, displayed neuroprotection. On the other hand, the administration of probenecid, a well-known Panx1 inhibitor, resulted in reduction of neuronal death and inflammasome activation in a rat model of ischemia [127]. Similarly, Cisneros-Mejorado and colleagues found that Panx1 channel inhibitors or Panx1 knockout mice showed a reduction of infarct volumes and improved motor function after ischemia/reperfusion injury in mice [128]. Freitas-Andrade and his colleagues suggested that the conflicting data from the literature may have arisen from the sex of the animals, or the steroid hormone status of the animal during the experiments [129]. They found that in female Panx1 knock out mice, infarct volume, astrogliosis, and microgliosis were significantly reduced, while such neuroprotection was not seen in male Panx1 knock out mice [129].

### 5.3. Traumatic Brain Injury

TBI impacts more than 10 million people worldwide every year, and it is responsible for 50,000 deaths and leaves more than 80,000 with permanent disabilities [130]. The pathophysiology of TBI largely consists of two distinct events: while primary injury occurs immediately after an impact to the head and results in necrotic death of glial cells, neurons, and blood vessels [131,132,133,134], secondary injury occurs days to weeks later, and it accompanies the sustained oxidative stress, excitotoxicity, and mitochondrial dysfunction caused by hypoxic-ischemic damage [130]. Neuroinflammation is a major constituent of the secondary injury, and if it is left unresolved, it can exacerbate the outcomes after TBI [135]. As mentioned above, DAPMS release upon trauma causes activation of astrocytes and microglia, which release a plethora of pro-inflammatory proteinases, cytokines, and chemokines into the parenchyma, leading to disruption of blood–brain barrier and infiltration of peripheral cells including leukocytes [136]. Depletion of neutrophils or blocking CD11d receptor improved neurological score, brain edema, and tissue damage after experimental TBI [137,138]. Moreover, genetic approaches that disrupted chemokine signaling in myeloid cells, including CCL2/CCR2 and CX3CL1/CX3CR1, showed acute protection after TBI in mouse models [139,140].

An important signaling pathway that is involved in chemotaxis is mediated by activation of purinergic (P2X and P2Y) receptors by ATP [141]. Pharmacological and genetic inhibition of various purinergic receptors reduced neuroinflammation, leading to improved outcomes in mouse models of spinal cord injury and TBI [142,143,144,145]. These results suggests that interdicting ATP release and the subsequent purinergic signaling can potentially serve as therapeutic targets. Using a controlled cortical impact (CCI) model to study brain trauma, our laboratory demonstrated a critical role of Panx1 channels in the development of neuroinflammatory response. We first showed that pharmacological blockade of Panx1 channels with Trovafloxacin significantly reduced macrophage infiltration and astrogliosis after TBI. These results correlated with an improvement in locomotor activity [146]. More recently, we showed that genetic deletion of Panx1 in myeloid cells, namely brain-resident microglia and infiltrating macrophages, led to an overall reduction in the infiltration of peripheral neutrophils and macrophages, as soon as 3 days after CCI injury [24]. Myeloid Panx1 conditional knockout mice also showed lower biomarkers of tissue damage, including a reduced level of α-II spectrin breakdown products and MMP9 [24]. Finally, the myeloid Panx1 conditional knockout mice showed improved motor function and blood–brain barrier dysfunction compared to control mice [24]. Overall, these data indicated that myeloid Panx1 channel activation play detrimental roles in brain trauma. Intriguingly, it has been recently proposed that serum Panx1 levels can serve as biomarker outcome for TBI patients [147]. Panx1 protein levels in the serum of TBI patients were significantly higher when compared to control individuals. Moreover, a negative correlation between Panx1 levels in the serum and favorable outcomes in the patients was found [147]. A concern with these findings is that Panx1 serum levels were almost as high as normal plasma levels of albumin. Thus, further studies are needed to validate these results in TBI patients.

## 6. Conclusions and Future Directions

In many CNS injuries, including spinal cord injury, TBI, and brain ischemia, extracellular ATP acts as a main danger signal to induce an orchestrated inflammatory response, which may lead to exacerbation of the injury [24,104,148]. Hence, ATP release via Panx1 channels may be a potential therapeutic approach for reducing inflammation in CNS injuries. Indeed, many studies thus far have shown the efficacy of Panx1 channel inhibitors (namely probenecid, mefloquine, carbenoxolone, and trovafloxacin) in animal models of cerebral ischemia [127], seizure [97], neuropathic pain [106], and TBI [24]. Undoubtedly, further investigations are needed to develop Panx1 inhibitors with minimal off-target effects, as many of the current inhibitors also target other large-pore channels, including the connexin hemichannels and CALHM1 channels [149]. Finally, the development of cell type-specific and inducible Panx1 knockout mice, followed by validation in human cells and humanized mice, will greatly aid in understanding of the role of Panx1 in neuroinflammation and, consequently, brain pathophysiology.

## Figures and Tables

**Table 1:** Summary of Panx1 function in different pathophysiologies.

Pathophysiology	Pharmacological Blockade of Panx1	Genetic Deletion	Outcome
Epilepsy	Probenecid and mefloquine	Global Panx1 KO	Blocked Ictal discharge and resistance to Kainic induced seizure (Dossi et al., 2018)
Spinal cord injury	^10^Panx, Mefloquine, Probenecid	Microglia Panx1 KO	Reduces Morphine withdrawal and joint pain by mechanical allodynia (Mousseau et al., 2018) (Burma et al., 2017)
Sciatic Nerve injury (Neuropathic pain)	Carbenoxolone and Trovafloxacin	Global Panx1 KO Myeloid Panx1 (Microglia and infiltrating monocytes) KO	Blockers reduced hypersensitivity to tactile and thermal stimuli Myeloid Panx1 KO did not reduced neuropathic pain (Weaver et al., 2017)
Ischemia (MCAO)	Probenecid	Global Panx1 KO Double KO of Panx1 and Panx2	Probenecid reduced neuronal death and inflammasome activation in rat model of ischemia.(Wei et al., 2015) Panx1 KO did not show neuroprotection but Double KO of Panx1 and Panx2 showed reduced neurological deficits and reduced infarct volume compared to WT in MCAO model (Bargiotas et al., 2011)
Ischemia/Reperfusion	Probenecid, mefloquine, Carbenoxolone	Global Panx1 KO	Blockers as well as Panx1 KO showed reduced infarct volume, neuronal and tissue damage and improved motor function. (Cisneros-Mejorado et al., 2015)
Traumatic brain injury	Trovafloxacin(TVX)	Myeloid Panx1 KO	TVX reduced macrophage infiltration and astrogliosis correlated with improvement in locomotor activity(Garg et al., 2018) Myeloid Panx1 KO showed improved motor co-ordination, memory outcomes, reduced tissue damage, less BBB leakage and less infiltration of leukocytes (Seo et al., 2020)
Experimental Autoimmune Encephalopathy	Mefloquine	Global Panx1 KO	Panx1 KO mice showed delayed onset of clinical signs of EAE and decreased mortality rate compared to WT mice Mefloquine (MFQ) reduced severity of acute and chronic EAE (Lutz, S.E., et al., 2013)
